# Brain Nuclei in the Regulation of Sexual Behavior, Peripheral Nerves Related to Reproduction, and Their Alterations in Neurodegenerative Diseases: A Brief Review

**DOI:** 10.3390/brainsci15090942

**Published:** 2025-08-29

**Authors:** María de la Paz Palacios-Arellano, Jessica Natalia Landa-García, Edson David García-Martínez, Jorge Manzo-Denes, Gonzalo Emiliano Aranda-Abreu, Fausto Rojas-Durán, Deissy Herrera-Covarrubias, María Rebeca Toledo-Cárdenas, Genaro Alfonso Coria-Ávila, Jorge Manuel Suárez-Medellín, César Antonio Pérez-Estudillo, María Elena Hernández-Aguilar

**Affiliations:** 1 Doctorado en Investigaciones Cerebrales, Universidad Veracruzana, Xalapa 91190, Veracruz, Mexico; arellanop.maripaz960@gmail.com (M.d.l.P.P.-A.); natalialanda15@gmail.com (J.N.L.-G.); edson_9810@hotmail.com (E.D.G.-M.); 2 Instituto de Investigaciones Cerebrales, Universidad Veracruzana, Xalapa 91190, Veracruz, Mexico; jmanzo@uv.mx (J.M.-D.); garanda@uv.mx (G.E.A.-A.); frojas@uv.mx (F.R.-D.); dherrera@uv.mx (D.H.-C.); rtoledo@uv.mx (M.R.T.-C.); gcoria@uv.mx (G.A.C.-Á.); josuarez@uv.mx (J.M.S.-M.); cesperez@uv.mx (C.A.P.-E.)

**Keywords:** sexual behavior, neuroendocrine regulation, major pelvic ganglion

## Abstract

Sexual behavior is a complex process in which the brain plays an active role. In the male rat, stimuli from the female are perceived through sensory receptors related to olfaction, hearing, vision, and the perigenital area, priming the individual for a sexual response. This process culminates with ejaculation and the deposition of semen into the uterine tract with the aim of achieving fertilization. The brain plays a fundamental role in both generating motivation and executing male sexual behavior. Meanwhile, the spinal cord, through the autonomic nervous system and the pelvic ganglion, transmits information to the reproductive organs, including the testes. Currently, there is extensive evidence demonstrating the involvement of various brain structures in the regulation of sexual behavior, as well as specific regions of the spinal cord involved in the control of ejaculation. For instance, the medial preoptic area (MPOA) has been shown to regulate the secretion of pituitary hormones, which in turn modulate the function of reproductive organs. Among these, testosterone production is particularly notable, as this hormone not only directly affects reproductive organs but also exerts a modulatory role on brain nuclei responsible for sexual behavior. Although there is a reciprocal regulation between the nervous and endocrine systems, it is important to note that the execution of sexual behavior also impacts peripheral structures, such as the major pelvic ganglion (MPG) and the testis, preparing the organism for reproduction. The purpose of this mini-review is to provide an overview of the main brain nuclei involved in the regulation of sexual behavior, as well as the spinal cord regions implicated in reproduction. Finally, we discuss how these structures may alter their function in the context of neurodegenerative diseases, aiming to introduce readers to this field of study.

## 1. Introduction

Male sexual behavior is a multifactorial process that requires the precise integration of both central and peripheral neuroendocrine mechanisms. This behavior begins with the perception of sensory stimuli processed by the central nervous system, particularly in regions such as the hypothalamus and limbic system, which are involved in the generation of sexual motivation and coordination of associated motor responses. Subsequently, the signal is transmitted through the spinal cord and autonomic pathways to the reproductive organs, enabling the execution of the sexual response [1,2,3].

At the central level, various brain regions are involved in regulating motivation and the execution of sexual behavior. Among these, the medial preoptic area, the amygdala, and the hypothalamus stand out for their integrative role in processing hormonal and sensory signals, which are then conducted to the spinal cord through descending projections [4,5].

Specialized structures such as the spinal bulbocavernosus nucleus (SNB) and the gastrin-releasing peptide (GRP) system modulate the activity of somatic motor neurons and excitatory interneurons, facilitating the motor execution of sexual behavior, especially during the erection and ejaculation phases. This spinal processing depends on hypothalamic signaling and hormonal regulation, allowing for the precise synchronization of genital motor responses according to the behavioral context [6].

On the other hand, the autonomic nervous system plays a crucial role in the modulation of visceral functions associated with male sexuality, acting through its sympathetic and parasympathetic components. These systems regulate processes such as penile vasodilation, accessory gland secretion, and seminal emission. In this context, the major pelvic ganglion (MPG) has emerged as a strategic node in the regulation of the male reproductive system, as it transmits information to structures such as the prostate, seminal vesicles, and testis, contributing to the maintenance of spermatogenesis and testosterone synthesis. This suggests a bidirectional interaction between central signals and peripheral neuroendocrine responses [7,8,9].

All the above constitute an integrative model that considers the functional interaction between the hypothalamic-spinal axis and peripheral structures such as the MPG. This approach proposes an innovative perspective on the neuroendocrine control of male sexual behavior, moving away from the traditional conception focused exclusively on the hypothalamic-pituitary-gonadal axis. Instead, it proposes a broader functional network that incorporates neurocentral, spinal, and peripheral mechanisms capable of modulating both sexual behavior and reproduction. A thorough understanding of this network is not only fundamental for the physiological research of sexual behavior, but also for the clinical approach to sexual and reproductive dysfunctions.

## 2. Materials and Methods

This review was conducted through a comprehensive and integrative analysis of the current literature on central and peripheral mechanisms related to male sexual behavior and their dysregulation in certain neurodegenerative pathologies. A broad search was performed in databases including PubMed, Google Scholar, SciELO, ScienceDirect, and Dialnet, using keywords such as testis, sexual behavior, neuroendocrine regulation, central nervous system regulation, sensory information, peripheral nervous system regulation, and major pelvic ganglion. Primary research articles, narrative and systematic reviews, meta-analyses, and studies employing animal models were included.

The selected studies provided insights into the hormonal regulation of male sexual behavior, focusing primarily on human research while also considering findings from animal models. Emphasis was placed on studies highlighting interactions among the brain, spinal cord, peripheral nervous system, major pelvic ganglion, and testis. Extracted findings were organized thematically into central and peripheral control mechanisms, underscoring their relevance to human pathophysiology.

## 3. Results

### 3.1. Central Circuits of Male Sexual Behavior: Central and Spinal Integration

Male rat sexual behavior is a complex and organized process involving a sequence of stereotyped motor patterns that are activated by sensory and hormonal stimuli, with the goal of achieving copulation and, potentially, reproduction. This behavior can be divided into four main phases, each of which involves intricate sensory processing and brain interactions.

Broadly, male rat sexual behavior consists of four primary phases: (a) Appetitive or sexually motivated phase: in this stage, the male actively seeks out a receptive female. During this phase, he performs behaviors such as sniffing the female’s genital area, chasing, grooming, and emitting vocalizations. This phase reflects their sexual motivation, regulated mainly by testosterone [10]; (b) Precopulatory phase: develops without genital contact. The male continues to pursue the female, which increases sexual motivation; (c) Copulatory or consummatory phase: clearly defined motor patterns are present, such as mating (the male positions himself on the female’s rump without penile intromission) and intromissions (when penetration does occur). The male may perform between 10 and 15 intromissions, interspersed with mating, until ejaculation occurs, and (d) post-copulatory phase: after ejaculation, the male enters a refractory period before restarting the sequence. This cycle can be repeated up to ten times, after which he reaches sexual satiety due to decreased motivation [11].

Sensory signals from the receptive female are integrated into various brain structures, allowing the male to properly execute sexual behavior until ejaculation is achieved [12]. In the appetitive phase, sensory stimuli inform the male about the receptive state of the female. The visual system provides information about her presence, the auditory system captures ultrasounds emitted by her, the somatosensory system is related to physical contact with the perineal region, and the olfactory system (the most relevant) detects pheromones emitted by the female in estrus, through the main olfactory epithelium and the vomeronasal organ. Olfactory signals activate structures such as the olfactory bulb and hypothalamus, facilitating sexual behavior [13,14,15,16,17], while the vomeronasal organ, besides being the specific site of pheromone detection, allows the male to identify the receptive female even if this structure is removed [18,19].

The **amygdala** also plays a fundamental role in regulating sexual behavior. Through its connections with the hypothalamus and the vomeronasal organ, it helps integrate the sensory and emotional signals that guide sexual behavior [12]. Thus, the medial nucleus acts as an integrating center for sensory stimuli, including sexual stimuli, and receives projections from the olfactory bulb and the vomeronasal organ. In turn, the amygdala projects fibers to the hypothalamus, organizing a multisynaptic network that regulates the motivation and execution of sexual behavior [14,19,20,21]. The hypothalamus is key in the integration of endocrine, autonomic, and behavioral information. The medial preoptic area (MPOA), an essential structure for the control of male sexual behavior, is located there [12,19,22,23,24,25]. It has been shown that mating and intromissions activate the MPOA, being this the area where male sexual behavior is initiated [26], so that the lesion of this area cancels such behavior [27]. These behaviors are not simple motor movements, but complex and directed actions, which require adequate orientation towards their goal, in such a way that the MPOA also regulates penile erection, a function in which the dorsomedial (DMHN) and ventromedial nuclei of the hypothalamus (VMHN) participate, the latter known as the “satiety center” for its role in inhibiting sexual behavior [28,29].

The bed nucleus of the stria terminalis (**BNST**) is a sexually dimorphic nucleus located in the medial basal forebrain. This heterogeneous structure regulates diverse functions, including mood, reward, and motivated behaviors such as drug seeking, feeding, and sexual activity. The BNST receives inputs from the olfactory bulbs and the medial and central amygdala, and projects to the hippocampus, ventral tegmental area, nucleus accumbens, lateral hypothalamus (LH), paraventricular nucleus, and the preoptic area—an essential site for the regulation of sexual behavior [30,31,32,33]. Through these connections, the BNST plays a central role in motivation and the initiation of consummatory behaviors [34].

Reciprocal projections between the BNST and other regions form an extended network that integrates chemosensory and hormonal signals relevant to sexual recognition. Within this circuit, BNST aromatase-expressing neurons process opposite-sex pheromonal cues transmitted via the medial amygdala, thereby enhancing mate preference and facilitating reproductive success [35].

The BNST is also critically involved in copulation and ejaculation. Lesions or dysfunction within this nucleus increase the number of intromissions before ejaculation, prolong inter-intromission intervals, and extend the post-ejaculatory refractory period. These findings indicate that ejaculation is not solely regulated at the spinal level, where the ejaculation center is located, but also under central control, in which the BNST plays a key role [35].

The **hippocampus**, part of the limbic system, is involved in spatial memory, learning, orientation, and emotional processing, such as stress. Its involvement in sexual behavior is limited. However, acute sexual experience is associated with increased neurogenesis and elevated corticosterone levels. Chronic experience normalizes these levels but maintains neurogenesis and reduces anxious behaviors [36,37], and it is proposed that these effects may be mediated by androgens, since the hippocampus possesses receptors for these hormones [38].

The **septal nucleus**, also known as the septal area, is a forebrain structure located in the basal part of the forebrain, close to the septum pellucidum, between the corpus callosum and the lamina terminalis. It plays a fundamental role in functions related to motivational states, emotional processing, and behavioral reward, including sexual behavior, which, being pleasurable, is reinforced and tends to be repeated in the future. In addition, the septal nucleus is involved in the control of autonomic and endocrine responses, in part through its connections with the hypothalamus via the stria medullaris of the thalamus and the fornix [39]. The involvement of the septal area in the regulation of sexual behavior has been evidenced by studies of lesions in the lateral and medial septal nuclei, which show opposite effects. Lesion of the lateral septal nucleus (LSL) increases intromission and ejaculation latencies and reduces the frequencies of mounting, intromission, and ejaculation. In contrast, lesion of the medial septal nucleus decreases mating and intromission latencies and increases pursuit and mating frequencies. These results suggest that both nuclei differentially modulate the execution of sexual behavior [40,41,42]. The involvement of the septum in the control of sexual behavior appears to be mediated by the noradrenergic system, as this region is richly innervated by fibers from the locus coeruleus and caudal brainstem [43].

Another structure involved and playing a central role in the regulation of the execution of sexual behavior is the medial preoptic area (**MPOA**). Evidence shows that this region is not only a neuroendocrine integration center, but also a key modulator of the sexual response, where several neural pathways and neurotransmitters are involved and where sensory and endocrine stimuli are integrated in the execution of copulatory behavior. In this sense, it has been shown that serotonergic fibers, originating mainly in the dorsal and medial nucleus of the raphe discharge this structure, establishing a fundamental pathway for serotonergic modulation on sexual behavior, exerting mainly an inhibitory effect on such behavior [44,45], using fluorescent immunohistochemical techniques, to describe the distribution of serotonergic fibers in the MPOA, demonstrated that these fibers tend to be denser in the central part of the MPOA. The presence of serotonergic fibers in the MPOA and its surrounding areas has not only been linked to the regulation of reproductive functions, but also to the release of gonadotropins to inhibit sexual behavior [44,46], suggesting that their modulatory activity is crucial in the organization and expression of sexual behavior by modulating the response to hormonal stimuli and behavioral expression. On the other hand, dopamine, which acts in mesolimbic pathways and in the hypothalamus, increases sexual motivation and facilitates the copulatory response [12,22]. Thus, the interaction between serotonin and dopamine involves a complex regulatory system, where serotonin exerts an inhibitory function, while dopamine promotes it. In addition, testosterone also interacts with serotonin and dopamine, adjusting the excitability and sensitivity of neurons in the MPOA and, therefore, facilitating the sexual response [30,44].

**Figure 1:**
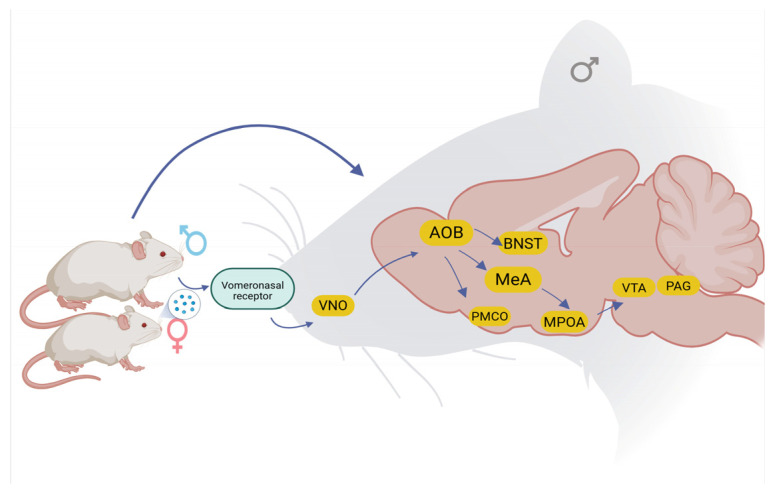
Schematic representation of nuclei involved in the regulation of sexual behavior. After sending the signal through the vomeronasal organ (VNO) to the accessory olfactory bulb (AOB), it passes to the medial amygdala (MeA), the bed nucleus of the stria terminalis (BNST), and the posteromedial cortical amygdala (PMCO). The MeA projects to the preoptic area (MPOA), which in turn projects to the ventral tegmental area (VTA) and the periaqueductal gray (PAG), thus coordinating sexual motivation, performance, and reward in male rats.

The **ventral tegmental area** (VTA) is a key structure in the regulation of sexual behavior, given its involvement in reward and motivation circuits [47]. This region is mainly composed of dopaminergic neurons and sends projections to several areas of the limbic system, including the nucleus accumbens, prefrontal cortex, and amygdala, playing a central role in the regulation of sexual behavior [47,48,49,50]. This structure contains dopaminergic neurons, although populations of GABAergic and glutamatergic neurons have also been identified, which modulate its function [51,52,53]. Dopamine is the main neurotransmitter in the VTA during sexual behavior, being at the center of motivational regulation and response to stimuli associated with sexual intercourse. During exposure to sexual stimuli, an increase in the activity of dopaminergic neurons has been documented [54,55], which facilitates the release of dopamine in the nucleus accumbens, reinforcing reproductive behaviors [52]. In parallel, endogenous opioids, enkephalins, and beta-endorphins are also involved in favor of the execution of sexual behavior [56]. Activation of μ-opioid receptors in the VTA leads to neuroplastic changes in dopaminergic neurons, including increased soma size and electrical activity, which contribute to the consolidation of this behavior [57]. Other neurotransmitters, such as serotonin and glutamate, also participate in the regulation of neuronal excitability in this structure and influence parameters such as latency and frequency of sexual activity [49,52]. The electrical activity of dopaminergic neurons in the VTA increases during the arousal phase and correlates with the dopamine released in mesolimbic areas related to reward, facilitating its execution in subsequent encounters [52,58]. In addition, there is evidence showing that hormonal modulation also influences behavioral response [59]. Testosterone, a steroid hormone essential in the regulation of male sexual behavior, increases dopaminergic activity in the VTA, favoring motivation and reinforcement of sexual behavior [60]. Evidence indicates that its presence increases the sensitivity of dopaminergic neurons and enhances the response to conditioned stimuli related to sexuality, thus facilitating the formation of memories related to sexual reward [32,61,62]. For its part, oxytocin plays a modulatory role in social perception and recognition, in addition to promoting dopamine release in the VTA during sexual interaction [63,64,65]. Its involvement in facilitating sexual motivation and bond formation is crucial, especially in the consolidation of reproductive behaviors [66,67]. In conclusion, the VTA is integrally involved in the regulation of sexual behavior through dopaminergic and opioid pathways, in which neuronal activity, modulated by hormonal mechanisms, facilitates neuroplastic changes and plasticity that support motivation, reward, and memory of sexual behavior (Figure 1) [52,58].

Sensory information from the perineal area, including tactile, thermal, and pressure stimuli, is captured by specialized receptors and transmitted to higher centers of the central nervous system. This information, once processed in regions such as the hypothalamus and brainstem, is sent by descending fibers to the spinal cord, particularly to the thoracic and lumbar segments involved in the regulation of seminal emission and erection. This process reflects a complex interplay between sensory afferents, central processing, and sympathetic and motor responses, which together underpin the functional context of sexual activity [68,69].

The lumbar spinal segments are of special interest because they contain neural circuits that, together with those in the thoracic and sacral regions of the spinal cord, are essential for the generation of male sexual responses, particularly erection and ejaculation [6]. Several nuclei relevant to these functions are in the spinal cord. In humans, Onuf’s nucleus is in the ventral horn of the sacral segments and is involved in muscle contraction during orgasm. In rats, its homologue is the spinal nucleus bulbocavernosus, located in the lower lumbar and upper sacral segments, which innervates the perineal striated muscles at the base of the penis [70,71,72].

In these lumbosacral segments are also located the neurons that originate the parasympathetic pelvic nerve, which innervates internal sexual organs such as the prostate and testes [73]. In addition, there are the spinotalamic neurons (LSt), located dorsolaterally to the central canal in lamina X between segments L3 and L4, which project to thalamic regions of the brain and are specifically associated with ejaculation, excluding behaviors such as riding or intromission. For this reason, they are referred to as the “spinal generator of ejaculation” and are characterized by expressing galanin [6,74,75]. These neurons project not only to the thalamus, but also to autonomic neurons located in the thoracolumbar and lower lumbar segments, which in turn innervate structures such as the prostate and seminal vesicles, in a circuit involving the pelvic and hypogastric nerves [73,76,77]. LSt neurons expressing GRP project to L5-L6 and S1 segments, as well as to the sacral parasympathetic nucleus. Galanin-expressing neurons send projections to the thalamus, from where the activation of the ejaculatory center is fed back through GRP neurons [6]. Although the descending pathways responsible for the control of emission and ejaculation are not completely understood, the MPOA has been identified as a key center for the execution of sexual behaviors such as mounting, intromission, and ejaculation. In this region is located the paraventricular nucleus (PVN), considered an autonomic regulation center due to its involvement in sexual activity and in penile erection [44,78,79,80]. This nucleus contains magnocellular neurons that synthesize oxytocin and project to the posterior pituitary, and oxytocinergic parvocellular neurons that project directly to the spinal cord, where they influence erection and sexual behavior [68].

The **hypothalamus**, in general, provides important descending projections to the spinal cord, particularly to lamina I of the dorsal horn and the columns of sympathetic and parasympathetic preganglionic neurons. At least five cytoarchitecturally distinct cell groups have been identified in the hypothalamus that contribute spinal projections, each with a predominance of specific neurotransmitters. In the PVN, a high percentage of projecting neurons contain arginine vasopressin (25–35%), oxytocin (20–25%), and metencephalin (10%). In the retrochiasmatic area, approximately 25% of the neurons contain α-MSH, whereas in the lateral hypothalamic tuberal area, this percentage reaches 100%. In the perifornical region, many neurons contain dynorphin (25%) or atrial natriuretic peptide (20%). Finally, in the dorsal hypothalamic area, between 55% and 75% of the neurons with spinal projection are dopaminergic, as demonstrated by immunostaining for tyrosine hydroxylase [8,81]. In addition to their role in modulating erection and ejaculation, these same lumbar and sacral segments are also indirectly involved in the regulation of spermatogenesis. This occurs through autonomic connections that modulate the endocrine and functional environment of the testes, influencing the function of Leydig and Sertoli cells, which are essential for sperm production and maturation.

In conclusion, male sexual responses are the result of a complex network of sensory afferents, specialized spinal circuits, and descending projections from brain centers such as the hypothalamus. These interactions enable the precise coordination of functions such as erection and ejaculation, which are essential for sexual behavior and male reproduction. The understanding of these mechanisms is fundamental for the clinical approach to sexual dysfunctions and for the advancement of knowledge in the neurobiology of reproduction.

### 3.2. Influence of Sexual Behavior on the Neuroendocrine Function of the Greater Pelvic Ganglion

The control of sexual behavior has been extensively studied at the brain level. However, the execution of this behavior serves the ultimate purpose of ensuring species survival through reproduction. A full understanding of its relevance also requires consideration of peripheral structures, such as the nerves emerging from the spinal cord that converge in the major pelvic ganglion (MPG), which regulates reproductive organs, including the prostate and testis [73,82,83]. The autonomic nervous system (ANS), through its sympathetic and parasympathetic divisions, regulates essential visceral functions during sexual intercourse, such as vasodilation of erectile tissue, contraction of accessory glands, and sperm emission [84].

These responses are modulated by descending pathways that originate in the brain and project to the lumbosacral spinal cord, where they synapse with preganglionic visceral neurons that, in turn, project to peripheral autonomic ganglia, including the MPG, which stands out as an integrative signaling center [85]. The MPG is a bilateral structure located in the pelvic cavity and, in rodents, is attached to the dorsolateral lobe of the prostate. It has a pyramidal shape, measuring approximately 2 mm wide by 4 mm long, and exhibits sexual dimorphism. It is composed of ganglion cells ranging from 100 to 1800 μm^2^ in the case of adrenergic neurons, and from 100 to 900 μm^2^ for cholinergic neurons. It also contains intensely fluorescent cells (SIF), of which there are two types differentiated by the size and morphology of their granules, as well as satellite cells, characterized by their simple morphology and their location surrounding the ganglion neuron, and Schwann cells [7,73,86].

Functionally, the MPG acts as a convergence point for two autonomic pathways: the hypogastric nerve (Hg), which originates from the inferior mesenteric plexus and the thoracolumbar segments T13–L2, with approximately 1600 predominantly noradrenergic sympathetic fibers; and the pelvic nerve (Pv), originating in the lumbosacral segments L6–S1, which carries approximately 5000 cholinergic fibers, organized into visceral and somatic branches [8,73]. These pathways converge in the MPG, forming a complex neuroanatomical network that modulates signals to various reproductive organs, such as the seminal vesicles, prostate, vas deferens, testicles, and penis [86,87].

For example, it has been observed that the execution of sexual behavior induces an increase in both the size of the ganglion and the neuronal soma. Furthermore, it has been reported that in the major pelvic ganglion (MPG) of subjects with sexual experience, neurons predominantly range from 300 to 900 μm^2^, compared to intact subjects, where neurons predominantly have an area below 300 μm^2^. These findings suggest that the execution of sexual behavior exerts a plastic effect on the MPG. In contrast, the absence of this neural control reduces both the size of the ganglion and the neurons, as well as the total number of neurons, highlighting the importance of neural regulation over this ganglion [7]. However, at the molecular level, no changes were found in the expression of receptors for adrenaline, noradrenaline, prolactin, and testosterone [88]. Future studies will investigate its effects on reproduction.

Another aspect of growing interest is the involvement of the MPG in testicular regulation, a function commonly attributed to the hypothalamic-pituitary hormonal axis. Gonadotropin-releasing hormone (GnRH), secreted by hypothalamic neurons located in the arcuate nucleus and medial preoptic area, stimulates the release of luteinizing hormone (LH), responsible for the biosynthesis and release of testosterone, and follicle-stimulating hormone (FSH), which acts on Sertoli cells to promote spermatogenesis [89]. This system operates through negative feedback: elevated testosterone levels inhibit the release of GnRH and gonadotropins, maintaining hormonal homeostasis [90].

Testosterone (T) exerts its effects on the central nervous system (CNS) by crossing the blood-brain barrier and binding to androgen receptors distributed in key regions for the regulation of sexual behavior, such as the medial hypothalamus, the medial preoptic nucleus (MPON), and the limbic system. Its conversion to dihydrotestosterone (DHT) or estradiol, by the action of the enzymes 5α-reductase and aromatase, respectively, regulates gene expression and neuronal excitability in these regions, modulating sexual motivation and motor patterns of copulation [1,3,91,92]. It also influences the frequency of erections, ejaculation latency, and the post-ejaculatory refractory period [89,90,93].

**Figure 2:**
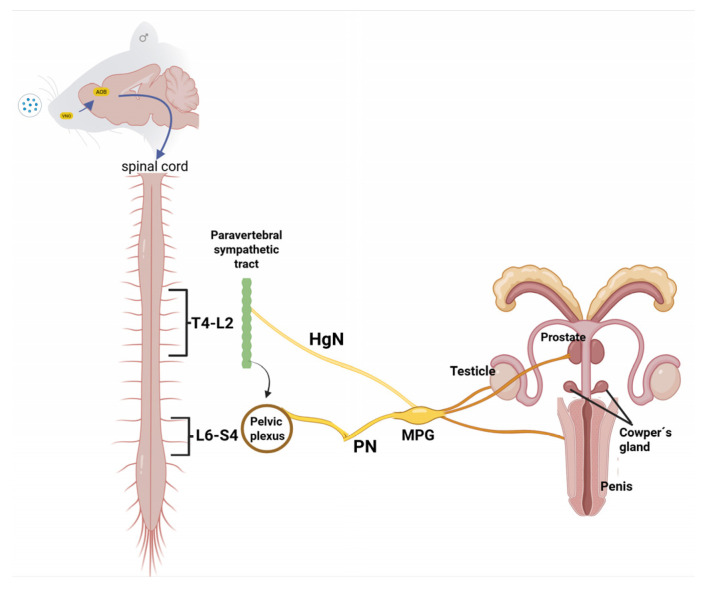
Central and peripheral integration of sexual behavior. Sexual stimuli processed in the brain activate descending projections to spinal circuits and peripheral structures such as the MPG, which integrates sympathetic and parasympathetic autonomic signals, modulating reproductive functions. VNO: vomeronasal organ, AOB: olfactory bulbs, HgN: hypogastric nerve, PN: pelvic nerve, MPG: great pelvic ganglion.

Recent research indicates that the testis receives both sympathetic and parasympathetic autonomic innervation from the major pelvic ganglion (MPG). Although further studies are required to fully elucidate the functional role of this innervation, evidence suggests that it contributes to the maintenance of testicular tissue, the development of spermatogenesis, and the synthesis of testosterone [8,9]. This relationship positions the MPG as a peripheral neuroendocrine modulator, potentially influencing local testosterone levels and androgen receptor activation (Figure 2) [89,90]. Notably, the absence of neural control prevents the typical increase in testosterone observed following sexual behavior. This effect becomes evident three days after denervation and is accompanied by a reduction in androgen receptor protein levels in the dorsolateral lobe of the prostate in male rats [8].

From an integrative perspective, it is important to consider the potential functional interactions between the MPG and the spinal nuclei involved in the motor execution of sexual behavior, particularly the spinal nucleus of the bulbocavernosus (SNB) and the gastrin-releasing peptide (GRP) system. Although the MPG does not project directly to the genital muscles, it forms synapses with SNB motor neurons in the L6 spinal segment, thereby modulating signals originating from the brain. Both the SNB and GRP systems receive projections from the paraventricular nucleus of the hypothalamus (PVN), serving as key intermediaries between hypothalamic processing and peripheral regulation of reproduction [6,91].

While direct synaptic connections have not been documented, it is plausible to propose a functional model in which afferent and efferent signals processed by the MPG influence the excitability of these circuits, given the ganglion’s close association with the L6 segment. In this framework, the MPG functions not only as an autonomic processing center but also as a component of a peripheral sensory-motor-neuroendocrine axis that links genital function to central and spinal control systems.

Although the direct impact of these changes on the testis has not yet been evaluated, it is hypothesized that they could increase neuronal electrical activity due to the action of hormones and neurotransmitters, which would be reflected in alterations in the cytoskeleton, connective tissue, and proliferation of SIF cells. These plastic effects induced by sexual behavior on the MPG appear to impact all reproductive organs, including the testis, where an increase in the area of the seminiferous tubules has been reported under these conditions. This makes sense, as this neuroendocrine circuit prepares the male to respond effectively to reproduction and ensure the survival of the species [9].

Thus, this integrative model proposes a broader view of the effects of male sexual behavior, in which the interaction between the brain, spinal circuits, and the MPG represents a key node for the coordination of hormonal, brain, and spinal signals. Understanding this network could have important clinical implications, particularly in sexual disorders of neural or spinal origin, autonomic neuropathies, or hormonal alterations, which could be causal factors in infertility.

### 3.3. Testosterone and Its Neurobehavioral Action

Testosterone is the main hormone involved in modulating male sexual behavior, both in terms of motivation and the execution of copulatory behavior. Its neurobehavioral action is exerted through genomic and non-genomic mechanisms, the former being mediated by androgen receptors (AR) present in brain regions such as the medial preoptic area (MPA), the medial hypothalamus, the medial amygdala, and the hippocampus [94]. Once in the brain, testosterone can act directly or be converted by the enzyme 5α-reductase type 2 into dihydrotestosterone (DHT) or into estradiol by the action of aromatase. DHT has a higher affinity for AR, and its binding induces receptor dimerization, translocation to the nucleus, and activation of androgen response elements (ARE) in DNA, leading to the transcription of genes involved in synaptic plasticity, neuronal excitability, and neuroeffector protein synthesis [91,95]. This hormone also regulates the expression of neurotransmitter receptors such as the dopamine D1 receptor, as well as dopamine transporters (DAT) in the ventral tegmental area and nucleus accumbens, facilitating mesolimbic dopaminergic neurotransmission related to sexual motivation [96]. Furthermore, it modulates the expression of neurotrophic factors such as brain-derived neurotrophic factor (BDNF), promoting dendritic arborization and the formation of new synaptic spines in structures such as the hippocampus and hypothalamus, which are involved in the emotional and physiological integration of sexual behavior [97]. On the other hand, non-genomic mechanisms involve the rapid activation of intracellular signaling pathways through membrane-associated androgen receptors, which activate second messengers such as protein kinase A (PKA), protein kinase C (PKC), or the PI3K/Akt pathway, which are signaling cascades that modulate neurotransmitter release, the excitability of hypothalamic neurons, and the activity of ion channels, rapidly and reversibly affecting sexual behavior [95,98]. The behavioral effects of testosterone have been extensively investigated in animal models, and it has been confirmed that castration produces a significant reduction in sexual behavior, while exogenous administration of testosterone or DHT restores copulatory parameters in a dose-dependent manner [4,93]. In addition, testosterone promotes the release of dopamine in the APOM during exposure to sexual stimuli, facilitating the initiation and maintenance of copulatory behavior [96].

Therefore, testosterone exerts control over male sexual behavior by integrating molecular mechanisms that regulate dopaminergic transmission, gene expression, and neuronal excitability in key brain regions of the motivational and reproductive systems.

Testosterone (T) has been shown to be the main modulator of male sexual behavior; however, other hormones also play a significant role in this regulation. One of these is oxytocin, a neuropeptide produced in the paraventricular (PVN) and supraoptic nuclei of the hypothalamus, which is released both centrally and peripherally. Centrally, oxytocin promotes the activation of neurons in the medial preoptic area and paraventricular nucleus, key regions for the initiation of erection and copulatory behavior [91]. On the other hand, peripherally, it acts on the myoepithelial cells of the vas deferens and seminal vesicles, promoting the ejaculatory reflex [77]. In addition, it modulates affective aspects of sexual behavior, such as post-copulatory social bonding and emotional reinforcement associated with the sexual experience [99]. Prolactin is also related to sexual behavior. Its plasma levels increase after ejaculation, which has led to the proposal that it acts as a negative regulator, inducing the post-copulatory refractory phase by temporarily decreasing sexual motivation [100]. Together, testosterone, oxytocin, and prolactin actively respond during sexual behavior and promote fertility.

### 3.4. Estradiol and the Role of Estrogens

A crucial aspect of the neuroendocrinology of male sexual behavior is the aromatization of testosterone to estradiol in the brain, a process mediated by the enzyme aromatase. This conversion occurs in specific regions of the brain, including the medial preoptic area (MPA), the medial amygdala, and the hypothalamus, where estradiol acts on estrogen receptors to modulate neuronal activity and behavioral expression associated with sexual behavior [101,102]. Various studies have shown that estradiol is essential for the restoration of copulatory behavior in castrated animal models. For example, the administration of aromatase inhibitors significantly reduces the motivation and execution of sexual behavior in rodents, even when circulating testosterone levels remain high [98]. Estradiol acts primarily through estrogen alpha (ERα) and beta (ERβ) receptors, which are expressed in neurons of the limbic system. Their activation regulates neuronal excitability, promoting the release of neurotransmitters such as dopamine and glutamate, and inducing changes in the gene expression of factors related to synaptic plasticity, such as brain-derived neurotrophic factor (BDNF) and synaptic proteins such as PSD-95 and synapsin I [103].

In addition, estradiol acts through intracellular signaling mechanisms, including the activation of pathways such as MAPK/ERK and PI3K/Akt, which are associated with long-term synaptic potentiation (LTP) processes, essential for learning and behavior consolidation [104]. These mechanisms allow estradiol to dynamically regulate the response of neural circuits involved in motivation, reward, and sexual behavior. Therefore, the aromatization of testosterone in the brain and the action of estradiol at specific neuroanatomical sites represent essential processes for the maintenance and expression of male sexual behavior.

### 3.5. Sexual and Reproductive Dysfunctions and Therapeutic Perspectives

Male sexual dysfunctions correspond to a significant alteration in any of the phases of the sexual response, including erection and ejaculation [105,106]. Alterations in neural centers, such as hypothalamic lesions or dysfunctions in dopaminergic neurotransmission, have been associated with disorders of sexual motivation [107]. Similarly, it has been reported that testosterone deficiency reduces the activation of the MPOA, compromising the motor execution phase of copulatory behavior [92].

Dysfunction of the GRP and SNB systems, whether resulting from spinal cord injury, peripheral neuropathy, or hormonal deficiencies, can lead to erectile dysfunction or failure of ejaculation, which in turn may indirectly prevent fertilization [105,106,107]. The MPG, as an integrative center for sympathetic and parasympathetic autonomic signaling, has emerged as a key player in the peripheral control related to reproduction. This ganglion modulates the activity of internal genital organs and erectile structures through autonomic innervation and has been proposed as a link between the central neuroendocrine system and peripheral testicular regulation [7,86,87]. The MPG has been identified as participating in the modulation of testosterone synthesis, testicular tissue maintenance, and spermatogenesis, acting as a neuroendocrine regulator [8,9,85]. It is important to note that infertility may be the result not only of direct damage to the testicle but also of the fact that this structure also innervates the prostate. Thus, the alteration in the innervation of this gland affects the production of the prostatic fluid necessary for sperm to survive in the female reproductive tract, because of the alteration of prostatic tissue [7].

From a therapeutic perspective, these findings open new avenues for the treatment of neurogenic sexual dysfunction. Therapeutic interventions aimed at spinal stimulation represent an innovative option for patients with erectile dysfunction secondary to spinal cord injury. On the other hand, the functional study of the MPG could allow the development of peripheral therapies that selectively modulate autonomic function without affecting the central axis, which could reduce systemic side effects, i.e., the neuroendocrine circuit that runs from the hypothalamus to peripheral effectors such as the MPG. This will allow for more accurate identification of the points of failure in different sexual dysfunctions, suggesting multifocal therapeutic approaches that integrate hormonal, neuropharmacological, neurostimulatory, and autonomic interventions.

### 3.6. Impact of Neurodegenerative Diseases on Male Sexual Behavior and Reproductive Function

Neurodegenerative diseases (NDs), such as Alzheimer’s disease, Parkinson’s disease, amyotrophic lateral sclerosis, and autism spectrum disorders, affect the nervous system by impairing neuronal function in both the central and peripheral nervous systems. This may be due to the involvement of both central and peripheral structures in the regulation of sexual desire, execution of sexual behavior, and even reproduction [108,109,110].

Although there is evidence that these diseases affect sexual behavior, they do not act in the same way, as the specific impact depends on the neural circuits involved, as will be described below.

#### 3.6.1. Alzheimer’s Disease

Alzheimer’s disease (AD) is a neurodegenerative disorder that causes progressive decline not only in cognitive functions but also in male sexual behavior and reproductive function. Clinical observations indicate that approximately 70% of patients report sexual indifference, and around 50% exhibit behavioral changes related to sexuality. These changes are linked to emotional or mood disturbances resulting from damage to the limbic system, which plays a central role in the regulation of sexual behavior [111].

This is supported by postmortem studies revealing early alterations in subcortical structures such as the hypothalamus and the limbic system, including the nucleus accumbens, amygdala, and hippocampus—all of which are closely associated with the modulation of sexual motivation [53,112]. As cognitive deterioration progresses, it impairs the ability to express, interpret, and contextualize sexual desire [113].

As the nervous system deteriorates, so do the neural connections responsible for regulating sexual response, affective bonding, and the spinothalamic pathways, which are part of the ascending somatosensory system and are essential for the conscious perception of sexual stimuli by enabling cortical integration of tactile and nociceptive information [114]. Moreover, recent studies have shown that patients with AD may experience erectile dysfunction, suggesting that this could be an intrinsic manifestation of neurodegeneration in the paraventricular nucleus, which is involved in penile erection. It may also result from damage to the spinal nucleus of the bulbocavernosus, which innervates the striated perineal muscles at the base of the penis, thus reducing erection and ejaculation [70,71,72,114,115,116,117].

From a hormonal perspective, a significant decline in testosterone levels has been documented, along with elevated luteinizing hormone (LH) and proinflammatory cytokines such as TNF-α. This disrupts the hypothalamic-pituitary-gonadal axis [118,119], resulting in hormonal deficiencies that affect sexual motivation—through dysregulation of mesolimbic dopaminergic circuits related to sexual reward—and reduce the neuroprotective role of testosterone against β-amyloid accumulation and synaptic loss [115,119].

Additionally, some studies have reported cognitive benefits from androgen therapy via its effects on dopaminergic pathways and reinforcement states, although its direct impact on sexual function still requires further evidence [115,116,117]. On a reproductive level, testosterone deficiency also impairs Leydig and Sertoli cell function, thereby reducing sperm production [118].

Importantly, AD may exacerbate the physiological decline associated with reproductive aging. Recent research has shown that the systemic oxidative stress and chronic inflammation characteristic of AD significantly interfere with spermatogenesis by inducing sperm DNA fragmentation, reducing motility, and altering sperm morphology, ultimately accelerating testicular decline [116,117,120]. These findings are supported by studies showing that β-amyloid 1–42 accumulation in the testes impairs sperm function, although further research is needed to clarify its clinical significance [121].

In summary, AD disrupts male sexuality through multiple mechanisms, including hormonal dysfunction, neurodegeneration of reflex and reward circuits, sperm impairment, and behavioral and emotional changes. The combined impact on sexual behavior and fertility leads to a substantial reduction in the patient’s reproductive health and quality of life [122].

#### 3.6.2. Parkinson’s Disease

Parkinson’s disease (PD) is a chronic neurodegenerative disorder that primarily affects the central nervous system and has a higher prevalence in individuals over the age of 60. It is estimated to affect approximately 1–3% of the population within this age group and is slightly more common in men than in women [123,124,125,126]. It has been documented that 70–80% of patients experience sexual dysfunctions such as reduced libido, erectile dysfunction, premature ejaculation, anejaculation, or, conversely, hypersexuality. This latter condition is often linked to poor impulse control and may be exacerbated by dopaminergic medication [127,128,129,130].

Animal studies have shown that the dopaminergic system is involved in sexual motivation, copulatory behavior, and genital reflexes. Central regions such as the MPOA and the paraventricular nucleus of the hypothalamus project to the hippocampus, brainstem, and spinal cord, coordinating sexual performance and reward [131,132]. Thus, excessive dopamine intake may contribute to disinhibited sexual behaviors in PD patients. Additionally, it was demonstrated that the Sry gene is also expressed in dopaminergic neurons of the nigrostriatal pathway. This gene regulates tyrosine hydroxylase (TH), a key enzyme in dopamine synthesis, and its cerebral expression is independent of gonadal hormones, suggesting a direct role in motor and dopaminergic function [133].

Furthermore, the degeneration of dopaminergic neurons in the substantia nigra and hypothalamus has been linked to hypospermatogenesis and reduced sperm production [134]. Although the precise connection between these brain regions and testicular function is not fully understood, evidence suggests the existence of alternative pathways to the hypothalamic-pituitary-gonadal axis, including hypothalamic projections to the spinal cord and autonomic innervation via the pelvic plexus or vagus nerve [87,135,136].

In summary, Parkinson’s disease negatively affects male sexual and reproductive function, although further research is needed to fully understand its reproductive implications.

#### 3.6.3. Autism Spectrum Disorder

Autism spectrum disorder (ASD) is characterized by early-onset behavioral disturbances. As individuals with ASD progress into adolescence and adulthood, sexual behavior naturally becomes part of their behavioral repertoire; however, it often manifests in ways that diverge from neurotypical expectations, reflecting the broader social and communicative challenges associated with the condition. The neural substrates underlying sexual behavior encompass a distributed network that includes the prefrontal cortex, orbitofrontal cortex, cingulate cortex, insula, cerebellum, amygdala, hypothalamus, and septum. Many of these regions exhibit structural and functional alterations in individuals with ASD. For example, the prefrontal cortex has been reported to contain an excess number of neurons, possibly due to altered neurogenesis or insufficient pruning [137]. The orbitofrontal cortex shows atypical connectivity, which may underlie difficulties in social decision-making and affective processing [138]. The cingulate cortex is frequently thinner in autistic individuals, particularly in its anterior subdivision, which is involved in emotional regulation and conflict monitoring [139]. The insula demonstrates hypoactivation during tasks involving interoception and social-emotional integration [140].

The hypothalamus, a key regulator of endocrine and sociosexual behaviors, exhibits dysregulation in oxytocin and vasopressin systems, with altered receptor expression and peptide release potentially contributing to impairments in bonding and sexual motivation [141,142]. The amygdala and septum show structural abnormalities, including differences in volume and cell density, which may influence emotional salience attribution and affiliative behavior [143,144]. Furthermore, the cerebellum, often hypoplastic in ASD, demonstrates a reduction in Purkinje cells and overall neuronal density, which has been associated not only with motor coordination deficits but also with atypical affective and cognitive processes [145].

Collectively, the neuroanatomical and functional alterations observed in ASD provide a biologically plausible substrate for the atypical patterns of sexual development, expression, and regulation frequently reported in this population. Despite this, the topic of sexuality in autistic individuals has historically been marginalized or misinterpreted, often overshadowed by a predominant research focus on core diagnostic domains such as social communication deficits, restricted interests, and repetitive behaviors. As a result, sexuality has remained an understudied—and at times deliberately avoided—aspect of autistic development and identity [146,147].

In recent decades, however, there has been a marked increase in scholarly attention to this issue, driven by a growing recognition of the central role that sexual health and identity play in overall well-being and quality of life. Emerging evidence demonstrates that the diversity of sexual expression within the autistic population is as broad as, and in some respects broader than, that observed in neurotypical individuals. Numerous studies have identified elevated rates of non-heterosexual orientations and gender nonconformity among autistic individuals, suggesting a higher prevalence of bisexual, asexual, pansexual, and non-binary identities in this group [148,149,150]. These findings challenge traditional assumptions and underscore the importance of adopting an inclusive, individualized, and non-stigmatizing framework when addressing sexuality in autism.

Moreover, individuals on the autism spectrum frequently encounter distinct barriers to sexual education, expression, and relationship development. These include social exclusion, limited access to appropriately tailored educational resources, and heightened vulnerability to misunderstanding or exploitation. Addressing these challenges necessitates a paradigm shift in both clinical and educational frameworks—one that affirms sexual and gender diversity, promotes neurodiversity, and equips autistic individuals with the knowledge, competencies, and autonomy required to engage in their sexual lives safely, confidently, and with dignity.

In conclusion, sexuality in the context of autism is a complex and multifaceted domain that demands sustained scientific inquiry and a holistic, person-centered approach. Advancing this field involves addressing deficits in social communication, enhancing access to inclusive sexual education, acknowledging sensory sensitivities, and actively confronting stigma and misconceptions. By doing so, we can contribute to enabling autistic individuals to lead fulfilling sexual and relational lives, in full respect of their autonomy, diversity, and fundamental human rights. Ultimately, the field must move beyond deficit-based models toward frameworks of empowerment—recognizing the inherent capacity of autistic individuals to experience, express, and enjoy their sexuality in authentic and meaningful ways.

#### 3.6.4. Multiple Sclerosis

Multiple sclerosis (MS) is a chronic autoimmune inflammatory disease that causes demyelination of neurons, leading to neurodegeneration of the central nervous system (CNS). It affects approximately 2.8 million people worldwide and has a global prevalence of 35.9 cases per 100,000 individuals [151].

MS impacts various physiological functions, including sexual behavior and reproduction. Although relatively few studies have addressed this topic, a reduction in pregnancy rates has been reported in individuals with MS [151]. Neuronal degeneration appears to interfere with the integration of genital stimuli. In patients with lumbosacral involvement, demyelination and axonal damage compromise the efficiency of the spinothalamic tract, contributing to genital response impairment and reduced sexual desire [152,153,154].

Sexual dysfunction affects 60–90% of men with MS [153,155,156]. This is largely due to direct damage to neural structures involved in sexual arousal and response. Demyelinating lesions in the thalamus, hypothalamus, and spinal cord impair genital reflexes and both sympathetic and parasympathetic signaling, leading to erectile and orgasmic dysfunction [157,158].

MS also produces secondary symptoms such as fatigue, spasticity, bladder and bowel dysfunction, neuropathic pain, anxiety, depression, and poor body image. These factors collectively contribute to decreased libido and overall sexual satisfaction [156,159]. Lesions in the thoracolumbar segments disrupt the spinothalamic tract and sacral reflex centers, reducing the perception of erotic stimuli and altering genital responses [77].

Amyotrophic lateral sclerosis (ALS), characterized by the progressive loss of upper and lower motor neurons, is also associated with sexual dysfunction. Although less extensively studied, degeneration of neurons in the spinal nucleus of the bulbocavernosus has been shown to impair perineal muscle function, affecting erection and ejaculation—even in the absence of cognitive or endocrine deficits [160].

From a hormonal perspective, men with MS often exhibit significantly reduced serum testosterone levels. This has been linked to damage in the arcuate nucleus and to dysfunction of the hypothalamic-pituitary-gonadal axis, contributing to decreased sexual desire [161,162,163]. Reduced levels of anti-Müllerian hormone (AMH) and decreased Leydig cell function have also been reported, suggesting testicular impairment with potential reproductive consequences [157,164].

Regarding male fertility, recent research has identified a causal link between MS and altered sperm morphology [165]. Nearly 50% of men with MS experience ejaculatory dysfunction, which negatively impacts both natural conception and the collection of semen samples for assisted reproductive techniques [156].

Despite these findings, significant barriers remain, including limited communication between patients and healthcare providers on sexual health, and a general lack of awareness about how MS and its treatments affect male fertility. These challenges underscore the urgent need for clinical protocols that integrate motor, hormonal, and reproductive aspects to improve the quality of life and sexual and reproductive health of men with MS [156,159].

## 4. Conclusions

Male sexual behavior represents a complex biological function, consisting of the dynamic interaction between central, spinal, and peripheral circuits. Testosterone has been considered the main hormonal modulator of this behavior. This hormonal modulation is functionally integrated with neuroanatomical circuits ranging from the medial preoptic area, amygdala, and hypothalamus to specialized spinal structures such as the SNB and GRP system, located in the lumbosacral segments.

At the spinal level, communication between somatic motor neurons and excitatory interneurons ensures the precise execution of the erection and ejaculation phases, which are highly dependent on hypothalamic signaling and androgenic regulation. For its part, the autonomic nervous system, through sympathetic and parasympathetic pathways, actively participates in the modulation of internal sexual organs. In this sense, MPG is now considered a neuroendocrine integration center in the male reproductive system.

This review proposes an integrative information that considers the interaction between the hypothalamic-spinal axis and peripheral structures such as the MPG, thereby redefining the traditional paradigm of male sexual control by including local feedback mechanisms and connected peripheral neuroendocrine processing centers. Therefore, understanding this functional network is essential not only for the physiological study of sexual behavior and reproduction. In this sense, targeted stimulation of spinal nuclei or selective modulation of the MPG could constitute innovative approaches to reproduction dysfunctions and open new perspectives for personalized interventions.

## Data Availability

No new data were created or analyzed in this study.
